# Describing the Profile of Diagnostic Features in Autistic Adults Using an Abbreviated Version of the Diagnostic Interview for Social and Communication Disorders (DISCO-Abbreviated)

**DOI:** 10.1007/s10803-019-04214-7

**Published:** 2019-09-07

**Authors:** Sarah J. Carrington, Sarah L. Barrett, Umapathy Sivagamasundari, Christine Fretwell, Ilse Noens, Jarymke Maljaars, Susan R. Leekam

**Affiliations:** 1 Department of Psychology, School of Life and Health Sciences, Aston University, Birmingham, B4 7ET UK; 2 School of Psychology, Wales Autism Research Centre, University of Cardiff, Cardiff, CF10 3AT UK; 3 St Cadoc’s Hospital, Aneurin Bevan University Health Board, Lodge Road, Caerleon, NP18 3XQ UK; 4 Integrated Autism Service, Unit 10 Torfaen Business Centre, Aneurin Bevan University Health Board, Panteg Way, New Inn, Caerleon, NP4 0LS UK; 5 Parenting and Special Education Research Unit, KU Leuven, Leopold Vanderkelenstraat 32, Bus 3765, 3000 Leuven, Belgium; 6 Leuven Autism Research (LAuRes), KU Leuven, Leopold Vanderkelenstraat 32, Bus 3765, 3000 Leuven, Belgium; 7 Child and Adolescent Psychiatry, UPC Z.org KU Leuven, Herestraat 49, 3000 Leuven, Belgium

**Keywords:** Autism spectrum disorder, DSM-5, Adult, Diagnosis

## Abstract

The rate of diagnosis of autism in adults has increased over recent years; however, the profile of behaviours in these individuals is less understood than the profile seen in those diagnosed in childhood. Better understanding of this profile will be essential to identify and remove potential barriers to diagnosis. Using an abbreviated form of the Diagnostic Interview for Social and Communication Disorders, comparisons were drawn between the profile of a sample of able adults diagnosed in adulthood and the profile of a sample of able children. Results revealed both similarities and differences. A relative strength in non-verbal communication highlighted a potential barrier to diagnosis according to DSM-5 criteria for the adult sample, which may also have prevented them from being diagnosed as children.

**Electronic supplementary material:**

The online version of this article (doi:10.1007/s10803-019-04214-7) contains supplementary material, which is available to authorized users.

Autism spectrum disorder (ASD) is a neurodevelopmental disorder affecting more than 1% of the population (e.g. Baird et al. 2006; Baron-Cohen et al. 2009; Brugha et al. 2011, 2016). Although the most recent publication of the Diagnostic and Statistical Manual for Mental Disorder (DSM-5; American Psychiatric Association 2013) recognises that symptoms may not become evident until later in life, individuals seeking diagnosis as adults may present with a different profile of features compared to those diagnosed as children.

Differences in the adult behavioural profile of ASD may present as reduced frequency of symptoms compared with childhood, as symptom frequency is known to decline with age (Charman et al. 2017; Fecteau et al. 2003; Fountain et al. 2012; Seltzer et al. 2004; Szatmari et al. 2015), particularly in those who have language ability and intellectual ability within the normal range (e.g. McGovern and Sigman 2005; Shattuck et al. 2007). However, little is known about the distinctiveness of the ASD profile in individuals presenting for diagnosis in adulthood. Studies examining the adult profile have examined patterns of behaviour in individuals who were already diagnosed in childhood or adolescence, not those who first presented for diagnosis in adulthood, and the results of such studies are equivocal. For example, a follow up study by Billstedt et al. (2007) of individuals diagnosed with ASD in childhood or early adolescence showed that difficulties in social interaction were more common in late adolescence and early adulthood than any of the other behaviours assessed, including both restricted and repetitive behaviours (RRBs) and non-verbal communication difficulties. In contrast a study by Shattuck et al. (2007) of adults first diagnosed as children or adolescents also found fewer RRBs in adults than younger individuals, but unlike Billstedt et al. (2007), found more non-verbal communication difficulties in adults than in children. While both of these studies suggest greater persistence of social interaction difficulties than RRBs into adulthood in individuals diagnosed during childhood or adolescence, they do not necessarily reflect the profile that could characterise those who do not seek a diagnosis until adulthood.

Understanding of the adult profile also needs particular consideration in terms of how behavioural features in adulthood align with diagnostic criteria. Detailed description can identify potential diagnostic markers distinctive in adults, and clarify concerns regarding potential barriers to obtaining a DSM-5 diagnosis of ASD (e.g. Wilson et al. 2013). There is evidence, however, that the most recently published diagnostic criteria—DSM-5 (American Psychiatric Association 2013)—may lack sensitivity for more able individuals (e.g. McPartland et al. 2012; Taheri and Perry 2012; see Kulage et al. 2014 for alternative finding) and adults (Wilson et al. 2013). Several studies have explored whether sensitivity could be improved by ‘relaxing’ the rules of DSM-5. DSM-5 states that individuals must have impairment in all three of the social-communication subdomains and in at least two of the four subdomains measuring RRBs. Relaxing the rules in one or both of these domains (for example requiring only two of the three social-communication subdomains) has been found to improve sensitivity in both children (e.g. Frazier et al. 2012; Mayes et al. 2013) and adults (Wilson et al. 2013).

In the current study, the behavioural profile associated with DSM-5 criteria was explored in a group of able adults first diagnosed in adulthood, many of whom completed the clinical interview without the presence of another informant. The inclusion of both self- and other-informant assessments was intended to better reflect the reality of the diagnostic process for adults and address a recognised gap in the literature (Mandy et al. 2018). Data were collected using the DSM-5 algorithm from an abbreviated version of the Diagnostic Interview for Social and Communication Disorders (DISCO; Leekam et al. 2002; Wing et al. 2002), which includes an algorithm to guide diagnosis according to DSM-5 (Carrington et al. 2014). These algorithms include items from the DISCO that map onto the behavioural subdomains described in the DSM-5 criteria, and incorporate the rules specified by DSM-5 (e.g. that an individual would need impairment in all three social-communication subdomains). By using an abbreviated form of the DISCO, it was possible to focus only on those behaviours considered most essential for the diagnosis of ASD (Carrington et al. 2014). The profile of behaviours in this adult sample was analysed in two ways. First, in terms of the percentage of items within each of the DSM-5 subdomains and second as the percentage of individuals meeting the threshold level that indicated the presence of the symptom being measured in that subdomain. These combined approaches, therefore, allowed investigation not only of the number of ‘difficulties’ in each behavioural subdomain, but also provided an indication of whether these difficulties were considered to present significant impairment. Finally, the percentage of individuals who exhibited behaviours within a DSM-5 algorithm previously identified as being highly discriminating for individuals with ASD (the ‘signposting set’; Carrington et al. 2015) was examined. The results from this adult sample were compared with the results previously published for children (Carrington et al. 2015; Carrington et al. 2014).

## Method

### Participants

Participants were 71 adults with clinically diagnosed ASD (mean age = 34.89 years SD 12.32; range 18–63; 46 male, 25 female). Fifty three had a clinical diagnosis of Autistic Disorder or Asperger Syndrome from a psychiatrist working in the National Health Service who used ICD-10 criteria for diagnosis. The remaining 18, recruited through the university recruitment register, also had a clinical diagnosis based on ICD-10 criteria but had been diagnosed by a range of different clinicians. All adults received their first diagnosis of autism as adults and this diagnosis was independent of the research interview. The IQs of participants were not assessed; however, none were registered with intellectual (learning) disability and all had verbal ability to self-report on their autism features in response to the interview, either alone or accompanied by a family member or friend. Thirty nine individuals were sole informants for the DISCO interview; additional information was requested from family members for questions where the interviewee had indicated that they did not know the answer, and was received in seven cases. The remaining 32 participants were accompanied by at least one other family member, spouse or partner, friend, social worker, foster carer, or advocate. The participant was always the primary interviewee, and generally answered all questions, unless they said they were unable to do so. The accompanying individual was always involved for questions relating to very early development and questions about early play and language, but were free to add information at any point during the interview. If the accompanying individual provided examples that the participant did not, indicating additional insight into social behaviour difficulties or evidence of learned strategies for example, then the accompanying individual’s testimony was used. It was very rarely a matter of disagreement between the informants, mostly a corroboration with the addition of missing information or further examples. However, if there was a disagreement then clinical judgment and observation was used.

The pattern of item responses were compared with child data from which the *DISCO DSM*-*5* algorithm (Kent et al. 2013) had been developed. Full details of the clinical and demographic details of the sample can be found in previous published reports for Sample 1 (Leekam et al. 2002; Wing et al. 2002) and Sample 2 (Maljaars et al. 2012). Only the higher ability (IQ of 70 or above) autistic child subsamples were selected for the analysis, comprising 35 children (34–131 months; 30 male) with a clinical diagnosis of *ICD*-*10* Childhood Autism or *DSM*-*IV*-*TR* Autistic Disorder. The original recruitment of samples had ethical approval from relevant regional ethics committees with the resulting datasets anonymised upon study completion. Use of these datasets in the current analyses was approved by Cardiff University’s School of Psychology Research Ethics Committee.

### Measures and Procedure

The Diagnostic Interview for Social and Communication Disorders (DISCO; Leekam et al. 2002; Wing et al. 2002) is a 320 item semi-structured interview schedule used with the parent or carer of an individual, or with the individual him/herself. The interview was not developed according to a specific set of diagnostic criteria; rather, its primary purpose is to elicit information relevant to the autistic spectrum in order to assist clinicians in their judgment of an individual’s level of development, disabilities, and specific needs. Needs are assessed in the context of difficulties experienced in the absence of structured support. The interview has good inter-rater reliability (Wing et al. 2002). It also contains diagnostic algorithms to inform clinicians’ decision-making. Good criterion validity has been found for the ICD-10/DSM-IV-TR algorithm (Leekam et al. 2002; Maljaars et al. 2012; Nygren et al. 2009). Good agreement has been reported with output on both the Autism Diagnostic Interview (ADI-R; Lord et al. 1994) and Autism Diagnostic Observation Schedule (ADOS-G; Lord et al. 2000) according to ICD-10/DSM-IV-TR criteria (Maljaars et al. 2012; Nygren et al. 2009). The existing breadth of items included in the DISCO meant that items could also be selected to create an algorithm for the DSM-5 criteria, which had high sensitivity and specificity (Kent et al. 2013).

A reduced DSM-5 item set (54 items; Carrington et al. 2014) has been derived from the full algorithm set through a process of statistical abbreviation. In brief, only items in the full algorithm that significantly discriminated between children with ASD and those with language impairment or intellectual disability were retained (see Fig. 3 for a full list of items). The threshold for each subdomain—i.e. the number of items required to indicate the presence of the behaviour being measured—was then calculated following the method used for the development of the full DISCO DSM-5 algorithm, and the abbreviated algorithm was tested using two independent samples. Like the full 85-item algorithm, the reduced 54-item DSM-5 algorithm has high sensitivity and specificity (for details, see Carrington et al. 2014). A further subset of 14 items within this reduced item set has been identified and published. This subset, referred to as the ‘signposting set’, has been found to be particularly highly discriminating for individuals with ASD (Carrington et al. 2015; Carrington et al. 2014). The ICD-10/DSM-IV-TR algorithm has also been abbreviated using the same statistical approach as for the abbreviation of the DSM-5 algorithm. The interview used in the current study (the DISCO Abbreviated) consisted only of items identified in the abbreviation of the ICD-10/DSM-IV-TR and DSM-5 DISCO algorithms; this is the first study in which these items have been used as a standalone interview.

In the DISCO—and DISCO Abbreviated—each item is coded according to level of impairment; most items were scored as present only if there was a ‘marked’ (severe) impairment, although some items were marked as present when there was a ‘minor’ impairment. For the DISCO Abbreviated, the algorithm settings regarding the application of marked or minor codes for each item were not changed from their original settings for the full algorithm (Kent et al. 2013; Wing et al. 2002). The DISCO includes both ‘current’ and ‘ever’ (lifetime) scores for each item. Where an item is endorsed currently it is also endorsed as an ‘ever’ code. Diagnosis is typically based on ‘ever’ scores, reflecting the neurodevelopmental nature of the condition. The focus in the current study was, therefore, on the lifespan or ‘ever’ codes^1^, except in the few cases where DISCO provided only a current scoring option (for example, non-verbal gestures; these items are marked with # in Fig. 3). This coding method provided consistency with previous publications (Carrington et al. 2015; Carrington et al. 2014; Kent et al. 2013) and facilitated comparison with the equivalent published data for children (Carrington et al. 2014; Leekam et al. 2002).

### Analysis

The broad profile of behaviour in children and adults was explored in line with the sub-domain and domain structure of the diagnostic criteria for DSM-5, represented by the DISCO algorithm (see Fig. 3 for the items in each subdomain for DSM-5). First, we analysed the percentage of items endorsed within each of the subdomains and domains in the algorithm. Second, we analysed the percentage of individuals meeting the total ‘cut-off’ or threshold for each subdomain/domain. The data for frequency of items within each domain/subdomain were not normally distributed (Shapiro–Wilk, p > .05), and the threshold data were categorical; consequently, Wilcoxon Signed Ranks and Friedman’s ANOVA were conducted as appropriate. Finally, the profile of behaviours observed in adults was also examined at the item-level. The percentage of individuals for whom each item was scored as ever having been present was plotted for the child and adult samples.

There were a number of differences between adult and child samples. For example only the adults were self-informants, while all children had parent-informants. Furthermore, the child sample included DISCO data that had initially been published in 2002, and represented, therefore, ‘older’ diagnoses. We, therefore, primarily focused on statistically analysing features and subdomain scores within each sample and did not draw direct statistical comparisons.

## Results

### Subdomain and Domain Level Analysis

**Fig. 1:**
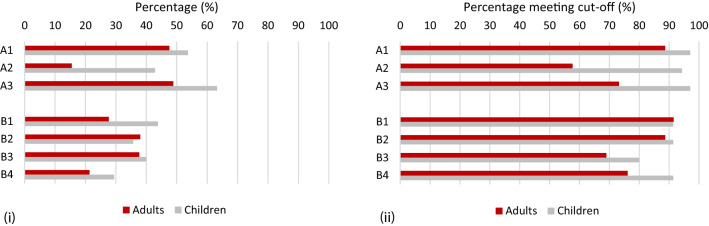
**i** The percentage of items of the abbreviated DISCO DSM-5 algorithm marked as ever present in each subdomain for children and adults; **ii** The percentage of children and adults who met the subdomain thresholds on the abbreviated DISCO DSM-5 algorithm. *A1* deficits in socio-emotional reciprocity, *A2* deficits in non-verbal communication behaviours used for social interaction, *A3* deficits in developing, maintaining, and understanding social relationships, *B1* stereotyped or repetitive motor movements, use of objects, or speech, *B2* insistence on sameness/inflexible routines/ritualised patterns of verbal/non-verbal behaviour, *B3* highly restricted, fixated interests that are abnormal in intensity or focus, *B4* hyper- or hypo-reactivity to sensory input/unusual interest in sensory aspects of environment

Within the social-communication domain for DSM-5, the subdomain with the highest percentage of items endorsed, both for adults and children, was that relating to developing, maintaining, and understanding social relationships (A3), with the lowest percentage in the subdomain relating to non-verbal communication (A2; Fig. 1i). Friedman’s ANOVA revealed a significant main effect of subdomain (children: $\chi_{(2)}^{2}$ = 19.24, p < .001; adults: $\chi_{(2)}^{2}$ = 73.49, p < .001). Figure 1 suggests that this effect was driven by the lower percentage of items in A2 compared with both A1 and A3. Given that five of the nine items in A2 measure current non-verbal gestures (see Fig. 3), the analyses were re-run including only the four items where the most severe manifestations of the behaviour were coded (‘ever’ codes). When just these four items were included for A2, the percentage of items scored in this subdomain was still lower than for either of the other social communication subdomains, and this effect was, again, more pronounced in adults (Fig. S1). Moreover there was still a significant effect of subdomain for both children ($\chi_{(2)}^{2}$ = 12.86, p = .002) and adults ($\chi_{(2)}^{2}$ = 42.14, p < .001).

Over 90% of children met the threshold in each of the A subdomains (Fig. 1ii). For adults, however, Friedman’s ANOVA revealed a significant main effect of subdomain ($\chi_{(2)}^{2}$ = 21.35, p < .001). The subdomain in which the most participants met criterion was A1, and the subdomain with the fewest was A2, which is consistent with the analysis of item frequency described above.

Within the RRB domain for DSM-5, the profile of behaviours observed for children and adults was somewhat different (Fig. 1i and ii). For children, the subdomain with the highest percentage of items was B1, relating to stereotyped or repetitive motor mannerisms, followed by B3, highly restricted, fixated interests that are abnormal in their intensity or focus. For adults, however, the subdomain with the highest percentage of behaviours was B2, insistence on sameness, inflexible routines, or ritualised patterns of verbal or non-verbal behaviour, closely followed by B3. Although the profile was different for each age group, analysis by item percentage showed a significant effect of subdomain for both children ($\chi_{(3)}^{2}$ = 12.51, p = .006) and adults ($\chi_{(3)}^{2}$ = 26.69, p < .001). Both age groups also had the smallest proportion of items in the subdomain measuring hyper- or hypo-reactivity to sensory stimuli (B4).

When the number of individuals meeting the subdomain threshold was considered, however, both children and adults had the lowest pass rate for B3 (restricted, fixated interests). For children, the pass rates in all other subdomains were equally high, while for adults, the subdomain in which most individuals met the threshold was B1. Friedman’s ANOVA revealed a significant effect of subdomain only for adults ($\chi_{(3)}^{2}$ = 17.82, p < .001), probably because of the near ceiling effect for children.

**Fig. 2:**
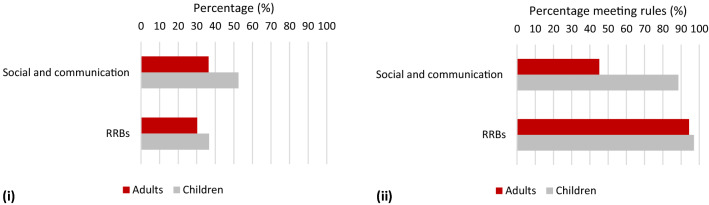
**i** The percentage of items within each domain marked as ever present in children and adults; **ii** the percentage of adults and children who met the domain rules based on ever scores

Finally, at the domain level, both children and adults scored on a significantly higher proportion of items in the social-communication (SC) domain compared with the restricted and repetitive behaviour (RRB) domain of DSM-5 (Fig. 2i; children: Z = − 3.89, p < .001; adults: Z = − 2.40, p = .017). To meet the threshold for each domain, DSM-5 specifies that all three A subdomains and at least two of the four B subdomains must be met. While a high proportion of children met the threshold for both the SC (88.6%) and RRB domain (97.1%), 54.9% of adults failed to meet threshold on at least one of the A subdomains (Fig. 2ii). By contrast, 94.4% of adults met threshold on at least two of the restricted and repetitive behaviour subdomains. Consistent with the percentage of behaviours endorsed in each domain, adults were significantly less likely to meet the thresholds for social communication difficulties than restricted and repetitive patterns of behaviour (Z = − 5.75, p < .001).

### Item Level Analysis (Fig. 3)

The frequency of individual items in the DSM-5 algorithm endorsed in adults was generally lower than in children. Nevertheless, there were several items with high frequencies in adults, particularly in the social-communication domain. Eight of the 25 social–communication items were present in 50% or more of adults. Four of these were in the subdomain relating to deficits in social emotional reciprocity, representing 44% of items in that subdomain, while the remaining four were in the subdomain relating to deficits in maintaining and understanding relationships (57.14% of items). Strongly endorsed social-communication behaviours in the adult sample included lack of awareness of others’ feelings (83.1%), does not interact with age peers (84.5%), no interest in age peers (71.8%), and lack of sharing of interests (73.2%), which were also highly frequent in the child datasets (85.7%, 82.9%, 71.4%, and 82.9% respectively). Another strongly endorsed behaviour in adults that was markedly less frequent in children was ‘does not share in others’ happiness’ (73.2%; children = 22.9%). None of the items relating to non-verbal communication were observed in more than 50% of the adult sample, while two of the nine items in this subdomain were present in 50% or more of the children.

**Fig. 3:**
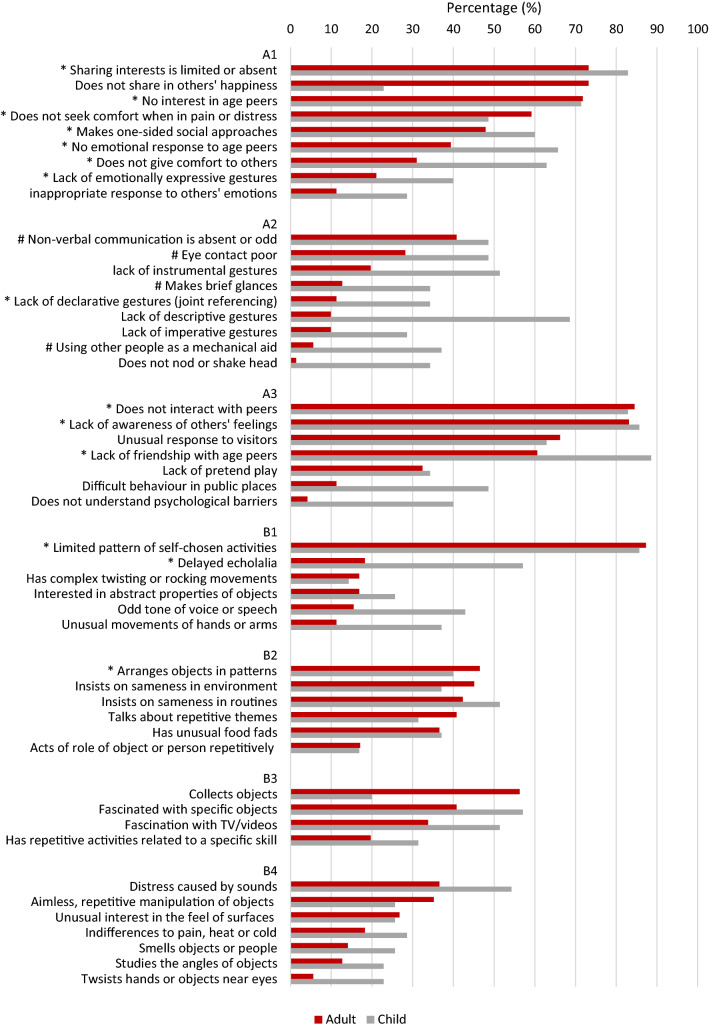
The percentage of adults and children for whom each DSM-5 item was ‘ever’ present. *Items in the ‘signposting’ set; #items for which only current scores are specified by the DISCO

Within the RRB domain, only two items were present in more than 50% of adults. These were ‘limited pattern of self-chosen activities’ in B1, endorsed by 87.3% of adults and 85.7% of children, and ‘collects objects’ in B3, which was endorsed by 56.3% of adults and just 20% of children. By contrast, in children six items across the RRB domain were endorsed in over 50% of the sample, including at least one from each subdomain.

### The Signposting Set

The 14 signposting items are indicated in Fig. 3*. While ten of these items were present in 50% or more of this sample of high ability children, seven were also present in more than 50% of the adult sample, indicating their diagnostic significance for adults. Indeed, all of the particularly strong social-communication items for adults identified above were signposting items. Six of these seven highly prevalent signposting items measured social-communication behaviours, three in A1 and three from A3. The remaining ‘signposting’ item present in more than 50% of adults was ‘limited pattern of self-chosen activities’. The mean number of ‘signposting’ items scored as ever present for the children and adults were 9.06 and 7.35 respectively.

## Discussion

Although many individuals do not receive a diagnosis of ASD until late adolescence or adulthood, relatively little is known about the profile of individuals who are first diagnosed with ASD as adults. In the current study, comparison of individuals diagnosed in adulthood and a sample of children revealed that the profiles in the two groups were similar overall, but with some distinctive differences.

The frequency of ASD features was generally lower in the adult sample, with the exception of a few items, such as ‘does not share in others’ happiness’ and ‘talks about repetitive themes’ (Fig. 3). Those items with higher frequency in the adult sample included behaviours less likely to be present in young children; for example, ‘does not share in others’ happiness’ is not coded in children younger than 7 years.

Both the adult and child samples exhibited more impairments in the social-communication domain of DSM-5 compared with the domain of restricted and repetitive patterns of behaviour (RRB). Moreover, within the social-communication domain, more difficulties were reported for both children and adults in socio-emotional reciprocity (A1) and in deficits in developing and maintaining relationships (A3) compared with non-verbal communication (A2). These effects were more pronounced in the adult sample, and were reflected both in terms of the percentage of items and in the percentage of individuals meeting threshold (Fig. 1). The relative lack of impairment in non-verbal communication has diagnostic significance. The DSM-5 criteria specify impairment in all three social-communication subdomains; the relatively good non-verbal communication skills within this adult sample could therefore mean that they do not qualify for a diagnosis of DSM-5 ASD. By contrast, difficulties in non-verbal communication are represented in two distinct subdomains within different domains of the ICD-10/DSM-IV-TR criteria; moreover, it would be possible to receive a diagnosis according to the ICD-10/DSM-IV-TR criteria without demonstrating impairment in either of these subdomains. Consequently, relatively good non-verbal communication skills in these areas would be less of a barrier to diagnosis according to ICD-10/DSM-IV-TR.

Evidence of difficulties in social interaction relative to both communication difficulties and RRBs supports findings from other studies involving adults, but who were diagnosed in childhood. For example, Billstedt et al. (2007) reported a higher proportion of difficulties related to social interaction compared with communication, RRBs, and emotional problems/maladaptive behaviour. In contrast, Shattuck et al. (2007) found that adults had more difficulties with non-verbal communication than younger individuals. Evidence of difficulties in non-verbal communication was not replicated in the current study, both when focusing on the most severe manifestation of behaviours (‘ever’ codes) and when examining the current profile (see Supplementary materials). These findings suggest that in a sample who were predominantly diagnosed as adults, non-verbal communication difficulties had never been as marked as difficulties with socio-emotional reciprocity and in developing and maintaining relationships.

Within the RRB domain, there was a slightly different profile of behaviour in the child and adult samples. Children had more behaviours in the subdomains relating to repetitive motor movements, use of speech, or objects (B1) and restricted, fixated interests (B3), both compared with the other subdomains and in comparison with adults. Adults had a higher percentage of behaviours in the subdomains relating to insistence on sameness/inflexible routines or rituals (B2) and restricted, fixated interests (B3) compared with both repetitive motor movements, use of speech, or objects (B1) and hyper- or hypo-reactivity to sensory input (B4).

The differing patterns of behaviour within the RRB domain are consistent with known developmental changes in two types of RRBs (e.g. Evans et al. 1997; Uljarević et al. 2017). Behaviours described in B2 and B3 have previously been described as higher-level RRBs, whilst behaviours described in B1 and B4 may be considered lower-level and more characteristic of children and lower-ability individuals (e.g. Prior and Macmillan 1973). Consistent with this argument, the child sample had a higher proportion of behaviours relating to stereotyped or repetitive movements (B1) than the adult sample. Moreover, the only subdomain in which adults had a higher frequency of items than children was B2 (insistence on sameness/inflexible routines or rituals). As such, the profile of RRBs seen in the child and adult samples are somewhat consistent with what might be expected for the two age-groups.

Despite these differences in the patterns of behaviour, the percentage of adults and children meeting the thresholds within each RRB subdomain (at least one behaviour present) was more similar. The greatest differences between the two samples were in B3 and B4. Although adults had a relatively high mean percentage of items in the subdomain relating to highly fixated interests (B3), this is the RRB subdomain with the highest threshold (see Fig. 1) and was, therefore, the subdomain in which the fewest adults met the threshold. Moreover, despite the relatively low proportion of behaviours relating to hyper- or hypo-reactivity to sensory input in both groups, the percentage of adults and particularly children who met the threshold for this subdomain was relatively high (75% for adults and 97.2% for children), indicating that the majority of individuals had at least one sensory symptom. Although this finding is consistent with Billstedt et al. (2007), the percentage of adults with at least one sensory symptom was lower in the current sample (75% compared with 93% as reported by Billstedt et al.). This finding may, again, be related to the nature of the sample, the majority of whom were diagnosed as adults, although this interpretation must be viewed with caution due to the limited size of the sample.

Given that the DSM-5 criteria require difficulty in just two of the four RRB subdomains, the potential implications of the differing RRB profiles for the child and adult samples for diagnosis are less striking initially than for the social communication domain. These differences do, however, highlight differences in the types of RRBs that might be expected in individuals presenting for diagnosis as adults. Rather than a focus on lower-level RRBs, these findings suggest that adults are more likely to present with difficulties relating to a lack of flexibility in their behaviour as demonstrated by adherence to routines and rituals, as well as fixated interests.

Exploration of the behavioural profile at the level of individual items identified some key behaviours that remained salient in the adult profile and could, therefore, be of significance for the identification of ASD in adults. While these were predominantly social–communication items, two additional behaviours from the RRB domain were present in over 50% of adults; these were a ‘limited pattern of self-chosen activities’ and ‘collects objects’. Some of the strongest items in adults were part of the ‘signposting set’, a set of items identified as being highly discriminating for children with ASD relative to those with language impairment or intellectual disability (Carrington et al. 2015). Although adults on average had fewer of the behaviours described in the signposting set than children, they still, on average, scored on over half of the items, indicating that this item set may still have potential in signposting when referral for ASD may be appropriate in adulthood.

When considering the potential implications of the findings from this study for the diagnosis of adults, it is important to consider whether the potential barriers to diagnosis that have been identified are from the DSM-5 criteria or from the interview used. Although the DISCO is a comprehensive interview designed to obtain a broad and detailed developmental history, the DISCO Abbreviated includes only a subset of items from the full interview. While those items were identified based on their predictive validity, the tool was developed and tested using a sample consisting predominantly of children. Moreover, those adults and adolescents included in the test samples for the abbreviated algorithm had been diagnosed in childhood or adolescence. It may, therefore, be necessary to identify further items for inclusion within the DISCO Abbreviated that may be more characteristic of individuals who do not seek diagnosis until adulthood, such as more items focusing on the types of RRBs that appear to be more characteristic of this adult sample. Nevertheless, the finding of fewer non-verbal communication difficulties in adults is consistent with the results reported by Billstedt et al. (2007), who used the non-abbreviated DISCO and also included items that were not part of the diagnostic algorithms. While further development of the DISCO Abbreviated and other interview tools will be important in supporting the diagnosis of adults, the DSM-5 specification that diagnosis is dependent on the presence of non-verbal communication difficulties may present a barrier for those seeking diagnosis in adulthood.

The majority of participants in the adult sample did not bring an informant with them for the interview with the DISCO Abbreviated. Although clinical guidelines for diagnosis recommend that a detailed developmental history should be conducted with an informant who has known the individual throughout their life and is able to comment on their behaviour, both currently and during childhood (National Institute for Health and Care Excellence 2011), such informants are not always available for adults seeking a diagnosis. Where they are able to attend, their memory of events during the individual’s childhood may lack detail. While these interviews can be done with the individual themselves, as they have been in the current study, they may lack detailed and accurate memories of their early development, or, for a subset of individuals, may not have detailed insight into their own difficulties. The inclusion of both self- and other-informant assessments in the current study was intended to better reflect the diagnostic process within adult services, and addresses a recognised gap in the literature (Mandy et al. 2018). Nevertheless, the limited number of participants within each of the two groups (with informant and self-informant) prevented formal comparison of the profile of behaviour described in these different interview approaches; however, the frequency with which items were endorsed by self-informants was comparable with those who brought an informant with them (see Fig. S5). Moreover, when total scores were calculated, using the algorithm, none of the overall subdomain or domain scores differed. Further investigation of potential differences, including comparison of self- and other-informant interviews for the same individual, will enable identification of behaviours that may be under- or over-reported by different informants.

Both the child and adult samples were diagnosed according to the ICD-10/DSM-IV-TR criteria. All individuals in the child sample had a diagnosis of Autistic Disorder or Childhood Autism. By contrast, the adult sample also included individuals with a diagnosis of Asperger Syndrome. While this diagnostic discrepancy could potentially account for differences observed in the behavioural profiles of the two groups, it may be a genuine reflection of the nature of these two samples. Evidence from children diagnosed with an ASD in the UK suggests that those with Asperger Syndrome are, on average, diagnosed later than those with autism (Crane et al. 2016; Howlin and Asgharian 1999). As such, a higher rate of Asperger-like presentations may be expected in those seeking diagnosis as adults. Nevertheless, the child sample selected for the current study was selected on the basis of a diagnosis of Autistic Disorder or Childhood Autism, and as such, it would be important to also draw comparisons between the adult sample, and children who fit the ICD-10/DSM-IV-TR criteria for Asperger Syndrome.

Many adults with ASD may have learned to camouflage certain areas of difficulty, for example, by learning to make eye contact during conversations and using pre-prepared social scripts. Moreover, many adults report learning to suppress repetitive motor mannerisms, particularly whilst interacting with others. While such techniques may have enabled them to function more effectively within complex social environments—thus potentially accounting for the delay in seeking support—the use of these techniques may also lead to individuals under-reporting their own difficulties, or to others underestimating the occurrence and impact of those difficulties. Social camouflaging is thought to be more pronounced in females than males with ASD (e.g. Hull et al. 2017; Lai et al. 2017), which could, therefore, impact on the relative rate of diagnosis for males and females. The DISCO interview, however, includes questioning to check for camouflaging and learned or rehearsed strategies. Coding is applied only with respect to what would be natural behaviour when no strategies are in place. Self-informants are also likely to be aware of their use of strategies to discuss in the interview. This might have mitigated against finding a gender difference in this study. While the current sample included too few females (n = 28) to draw meaningful comparisons with males (n = 52), a growing body of research has been focusing on the so-called ‘female profile’ of ASD. For example, it has been suggested that in young children, repetitive behaviours or circumscribed interests around dolls may be misinterpreted as pretend play, while older females with ASD may exhibit apparently benign repetitive behaviours, such as constantly reading a specific set of books to the detriment of social interaction (Halladay et al. 2015). Such behaviours may be undetected, potentially contributing to reports of lower levels of repetitive behaviours in females than males with ASD (e.g. Mandy et al. 2012). As such, it will be important to identify behaviours characteristic of adult females with ASD to facilitate their diagnosis and subsequent access to appropriate support.

The current study is the first use of an abbreviated form of the DISCO as a standalone clinical interview, and thus represents an important step in facilitating diagnosis within a more clinically feasible time frame. The findings indicate both similarities and differences between the child and adult profiles of ASD, with the differences highlighting potential barriers to diagnosis according to DSM-5 criteria for higher ability adults, which may also have prevented them from being diagnosed as children. A key difference between the child and adult samples was the lower rate of non-verbal communication difficulties in the adult sample. This relative strength in adults could represent a particular barrier to diagnosis according to the DSM-5 criteria that would not be as pronounced for the ICD-10/DSM-IV-TR criteria. In cases where a lack of impairment in non-verbal communication is the only contraindication of an ASD diagnosis, it may be prudent, therefore, to consider ‘relaxing’ the rules for the social-communication domain of DSM-5 to require impairment in just two of the three subdomains. Furthermore, although the current study has found that a ‘signposting set’ of items with high predictive validity in children also has the potential to signpost diagnosis in adults, it will be important to identify behaviours that are more characteristic of this population of able individuals with ASD, who may be missed in childhood, to facilitate their diagnosis and access to support at an earlier age.

## Electronic supplementary material

Below is the link to the electronic supplementary material.

Electronic supplementary material 1 (PDF 359 kb)

## Footnotes

1 Analysis of the ‘current’ profile is presented in the supplementary materials
