# Supplementary material for: Describing the Profile of Diagnostic Features in Autistic Adults Using an Abbreviated Version of the Diagnostic Interview for Social and Communication Disorders (DISCO-Abbreviated)
Source: J Autism Dev Disord. 2019 Sep 7;49(12):5036–46. doi: 10.1007/s10803-019-04214-7 (PMC6841916; doi:10.1007/s10803-019-04214-7)

Describing the profile of diagnostic features in autistic adults using an abbreviated version of the Diagnostic Interview for Social and Communication Disorders (DISCO-Abbreviated).

Journal of Autism and Developmental Disorders

Supplementary materials: The supplementary materials include the results for analysis of the profile of behaviours scores as being currently present, and a comparison of the percentage of adults who were accompanied and unaccompanied for whom each DSM-5 item was 'ever' present.

Sarah J. Carrington[1,2], Sarah Barrett[2], Umapathy Sivagamasundari[3], Christine Fretwell[4], Ilse Noens[5,6], Jarymke Maljaars[5,6,7], Susan R. Leekam[2]

[1]Department of Psychology, School of Life and Health Sciences, Aston University, Birmingham, B4 7ET; [2]Wales Autism Research Centre, School of Psychology, University of Cardiff, UK, CT10 3AT; [3]Aneurin Bevan University Health Board, Llanfrecha Grange Hospital, Cwmbran, Torfaen, NP44 8YN; [4]Aneurin Bevan University Health Board, St Cadoc's Hospital, Lodge Road, Caerleon, NP18 3XQ; [5]Parenting and Special Education Research Unit, KU Leuven, Leopold Vanderkelenstraat 32, Bus 3765, 3000 Leuven, Belgium; [6]Leuven Autism Research (LAuRes), KU Leuven, Leopold Vanderkelenstraat 32, Bus 3765, 3000 Leuven, Belgium; [7]Child and Adolescent Psychiatry, UPC Z.org KU Leuven, Herestraat 49, 3000 Leuven, Belgium

Corresponding author

Email: s.carrington@aston.ac.uk; Tel: +44 121 204 4322; Fax: +44 121 3696

Supplementary materials

DSM-5 current results

Subdomain and domain level analysis

Consistent with the "ever" codes, in the social communication subdomain, both children and adults had the highest percentage of items in A3 and the lowest in A2 (Figure S2(i)). Friedman's ANOVA revealed a significant effect of subdomain for both children ($\chi^2_{(2)}$=6.49, p=.039) and adults ($\chi^2_{(2)}$=80.89, p<.001). When the percentage of individuals meeting threshold in each subdomain was considered (Figure S2(ii)), the pass rates in each subdomain were lower for "current" scores. The pattern of the results for adults was comparable with the "ever" score; the highest pass rate was in A1 and the lowest in A2, and there was a significant effect of subdomain ($\chi^2_{(2)}$=19.60, p<.001). In children, however, the highest pass rate was seen in A1 and the lowest was in A3, although there was no significant variation across the subdomains.

Within the RRB domain, the pattern of behaviours was consistent with the "ever" scores for both adults and children, and there was once more a significant effect of subdomain for both groups (children: $\chi^2_{(2)}$=11.15, p=.011; adults: $\chi^2_{(2)}$=16.80, p=.001). Similarly, the percentage of individuals meeting threshold in each of the subdomains was consistent with the "ever" scores, again with a significant effect of domain only for the adults ($\chi^2_{(2)}$=18.43, p<.001).

At the domain level (Figure S3), as for "ever" scores, both adults and children had a significantly higher percentage of the social-communication difficulties as compared with

RRBs (children: z=-4.05, p<.001; adults: Z=-2.64, p=.008). Both children and adults were significantly less likely to meet threshold for the social-communication domain compared with the RRB domain (children: z=-3.32, p=.001; adults: Z=-5.67, p<.001). For the child sample, this pattern is different to that seen for their "ever" scores, where the percentage of individuals meeting threshold in the two domains was similarly high.

Item level analysis (Figure S4)

As for the "ever" scores, the frequency of individual items in the DSM-5 algorithm, currently endorsed by adults was generally lower than in children. Six of the 25 social communication items were present in more than 50% of adults, three each from the subdomains relating to social emotion reciprocity and deficits in maintaining and understanding relationships. While items relating to a lack of interest in age peers and a lack of friendship with age peers were present in 71.8% and 60.6% of adults respectively when "ever" scores were considered, neither difficulty was currently reported for more than 50% of adults. Within the RRB domain, no items were endorsed as being currently present by more than 50% of adults.

Comparison of the percentage of adults who were accompanied and unaccompanied for whom each DSM-5 item was 'ever' present (Figure S5)

Mann-Whitney comparisons corrected for multiple comparisons (Bonferroni correction, p<.001) revealed that there were no items that were endorsed by a significantly different number of individuals in the two groups. Two items approached significance, being endorsed by more individuals in the accompanied group (Lack of descriptive gestures: U=487.5, p=.002; acts role of object or person repetitively: U=496.5, p=.023), however, neither survived the correction for multiple comparisons.

Figures

Fig. S1 The percentage of items within each social-communication subdomain marked as ever present in children and adults, when only the non-verbal communication items were included in which 'ever' codes could be scored

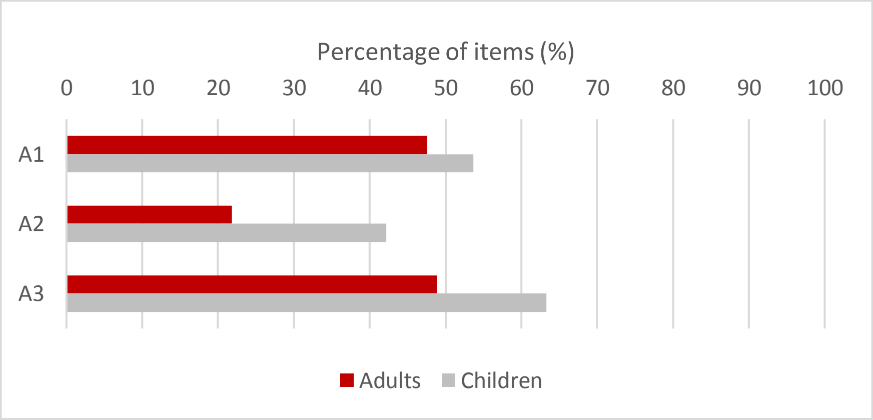

Fig. S2 (i) the percentage of items within each domain marked as currently present in children and adults; **(ii)** the percentage of adults and children who met the domain rules based on "current" scores

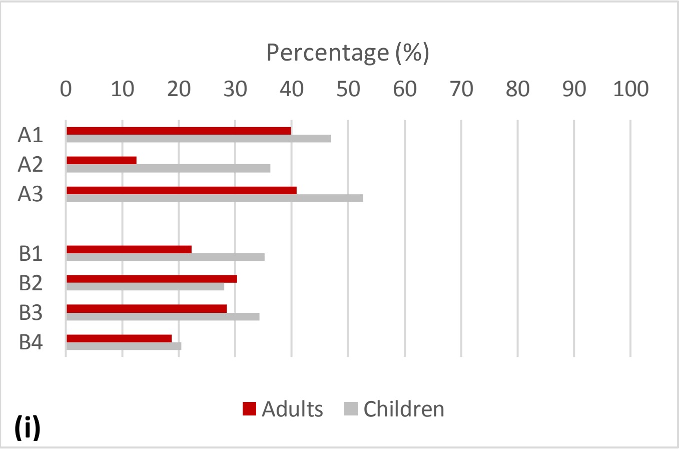

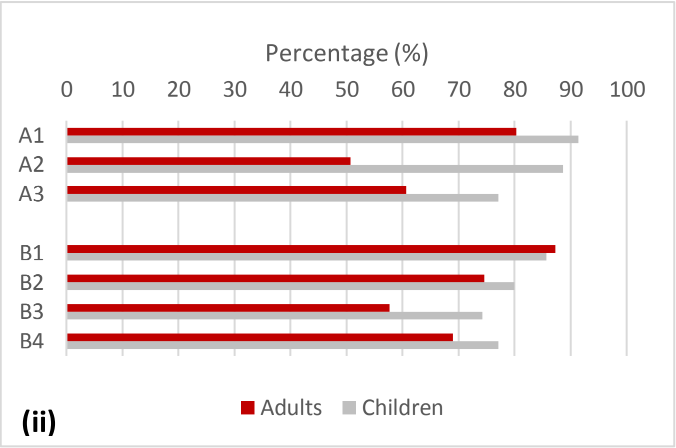

Note. A1=deficits in socio-emotional reciprocity; A2=deficits in non-verbal communication behaviours used for social interaction; A3=deficits in developing, maintaining, and understanding social relationships; B1=stereotyped or repetitive motor movements, use of objects, or speech; B2=insistence on sameness/inflexible routines/ritualised patterns of verbal/non-verbal behaviour; B3=highly restricted, fixated interests that are abnormal in intensity or focus; B4=hyper- or hypo-reactivity to sensory input/unusual interest in sensory aspects of environment

Fig. S3 (i) the percentage of items within each domain marked as currently present in children and adults; **(ii)** the percentage of adults and children who met the domain rules based on "current" scores

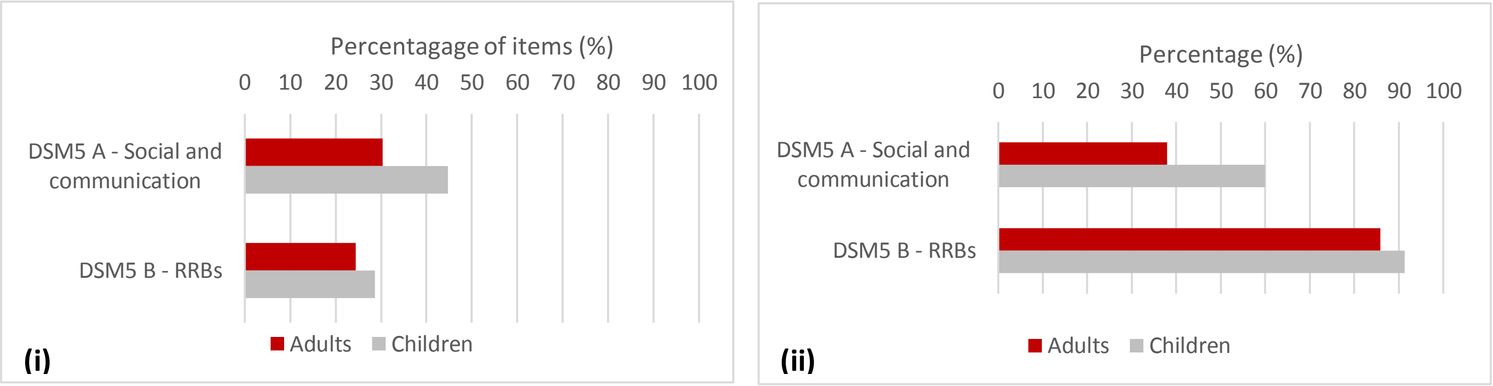

Fig. S4 The percentage of adults and children for whom each DSM-5 item was currently present. * denotes items in the 'signposting' set

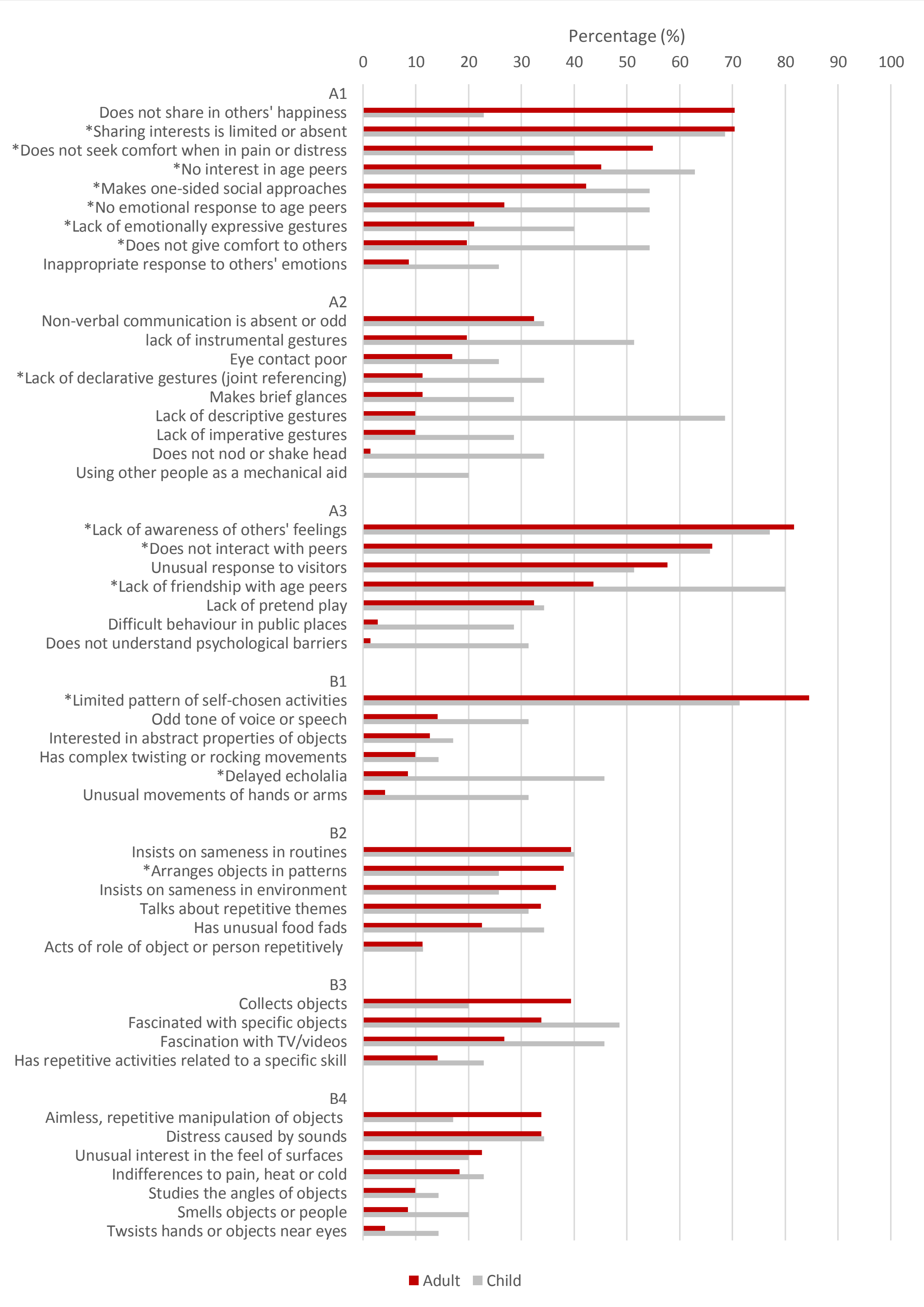

Fig S5 The percentage of accompanied and unaccompanied adults for whom each DSM-5 item was 'ever' present. * denotes items in the 'signposting' set

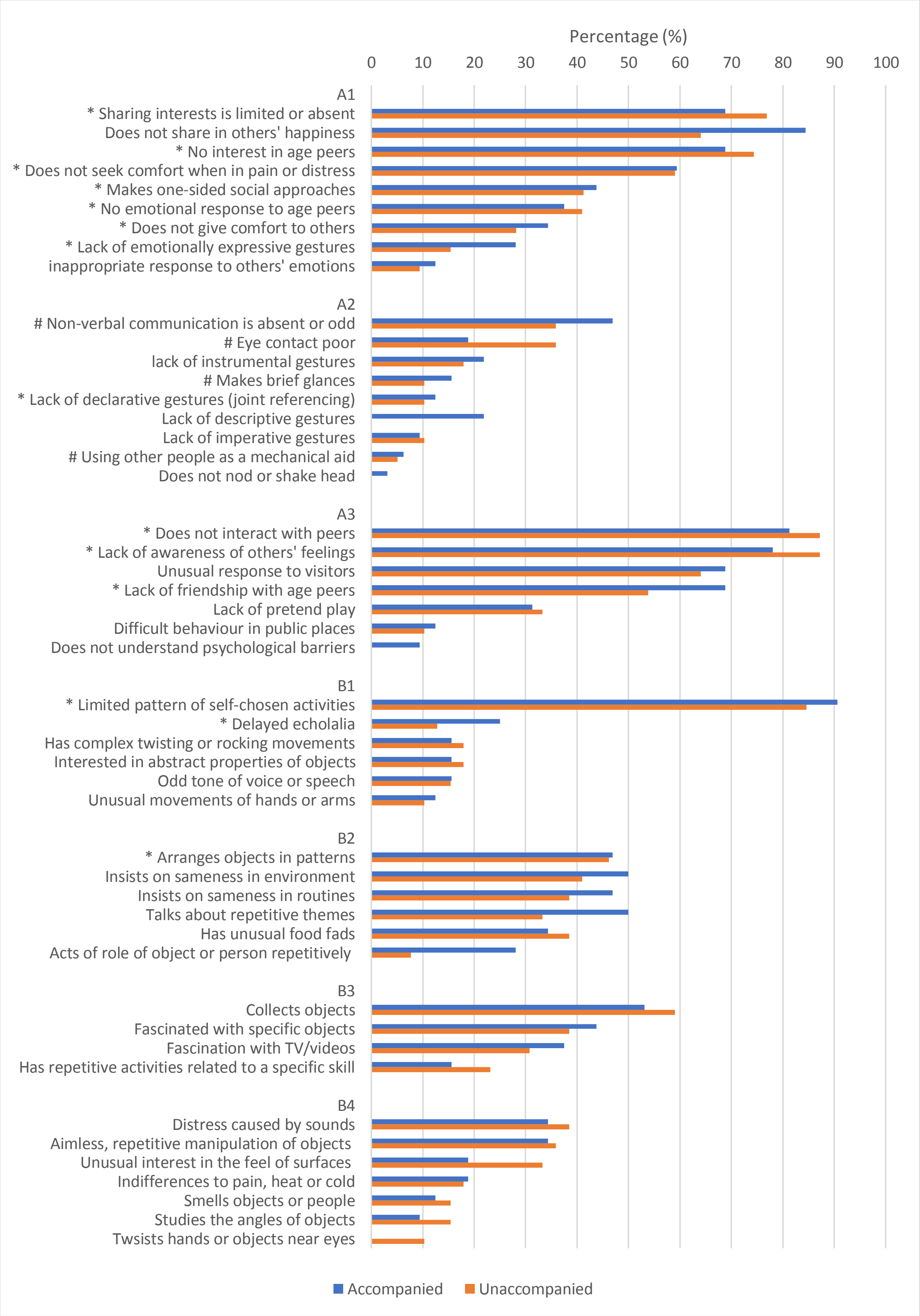

Supplement: Supplementary file 1 — Electronic supplementary material 1 (PDF 359 kb) [file 10803_2019_4214_MOESM1_ESM.pdf]
